# Narrowing the Digital Divide: Framework for Creating Telehealth Equity Dashboards

**DOI:** 10.2196/57435

**Published:** 2024-09-04

**Authors:** Michael J Luke, Sansanee Craig, Suzinne Pak-Gorstein, Marlíse Arellano, Jessica Zhang, S Margaret Wright, John Chuo, Philip V Scribano

**Affiliations:** 1 Children's Hospital of Philadelphia Philadelphia, PA United States; 2 Clinical Futures Children's Hospital of Philadelphia Philadelphia, PA United States; 3 Leonard Davis Institute of Health Economics Perelman School of Medicine University of Pennsylvania Philadelphia, PA United States; 4 PolicyLab Children's Hospital of Philadelphia Philadelphia, PA United States; 5 Department of Biomedical Health Informatics Children's Hospital of Philadelphia Philadelphia, PA United States; 6 Department of Pediatrics Perelman School of Medicine University of Pennsylvania Philadelphia, PA United States; 7 Department of Pediatrics School of Medicine University of Washington Seattle, WA United States; 8 Boston Children's Hospital Boston, MA United States; 9 Children's Mercy Kansas City University of Missouri-Kansas City School of Medicine University of Kansas School of Medicine Kansas City, MO United States

**Keywords:** telehealth, equity, dashboard, data, framework, televisit, healthcare, disparity, disparities, clinician, clinicians, informaticist, informaticists, researcher, researchers, pediatric, pediatrics, health system, health systems, dashboards, access to care, data source mapping

## Abstract

Telehealth presents both the potential to improve access to care and to widen the digital divide contributing to health care disparities and obliging health care systems to standardize approaches to measure and display telehealth disparities. Based on a literature review and the operational experience of clinicians, informaticists, and researchers in the Supporting Pediatric Research on Outcomes and Utilization of Telehealth (SPROUT)–Clinical and Translational Science Awards (CTSA) Network, we outline a strategic framework for health systems to develop and optimally use a telehealth equity dashboard through a 3-phased approach of (1) defining data sources and key equity-related metrics of interest; (2) designing a dynamic and user-friendly dashboard; and (3) deploying the dashboard to maximize engagement among clinical staff, investigators, and administrators.

## Telehealth Equity

The COVID-19 pandemic catalyzed a surge in telehealth adoption [1,2]. However, disparities in access to and adoption of digital health care persist among Black, Hispanic, public-insured, low-income, and rural populations [3,4]. This “digital divide” risks worsening health disparities in these populations [5]. As such, Crawford and Serhal [6] created the Digital Health Equity Framework (DHEF) to guide the equitable design and implementation of future digital health interventions. The DHEF takes into consideration, how individuals’ sociocultural and economic contexts influence intermediate factors, such as environmental stressors and health behaviors, which then drive the digital determinants of health (eg, acceptability of or access to digital health and digital health literacy) at the root of these disparities.

While health systems can use the DHEF to implement equity-minded telehealth strategies, understanding and bolstering the quality of the digital infrastructure within the communities they care for are critical steps to ensuring equitable access to telehealth [7]. Unfortunately, digital analytics are still lacking in understanding patterns of use for those underserved by technology infrastructure. Dashboards that showcase key performance indicators in real-time have become valuable tools to track health care access, understand disparities, and apply interventions. Yet, there are no consensus guidelines for the creation of telehealth-specific equity dashboards, which can apply the nuanced considerations for telehealth equity outlined through the DHEF to existing standards for data monitoring.

**Figure 1:**
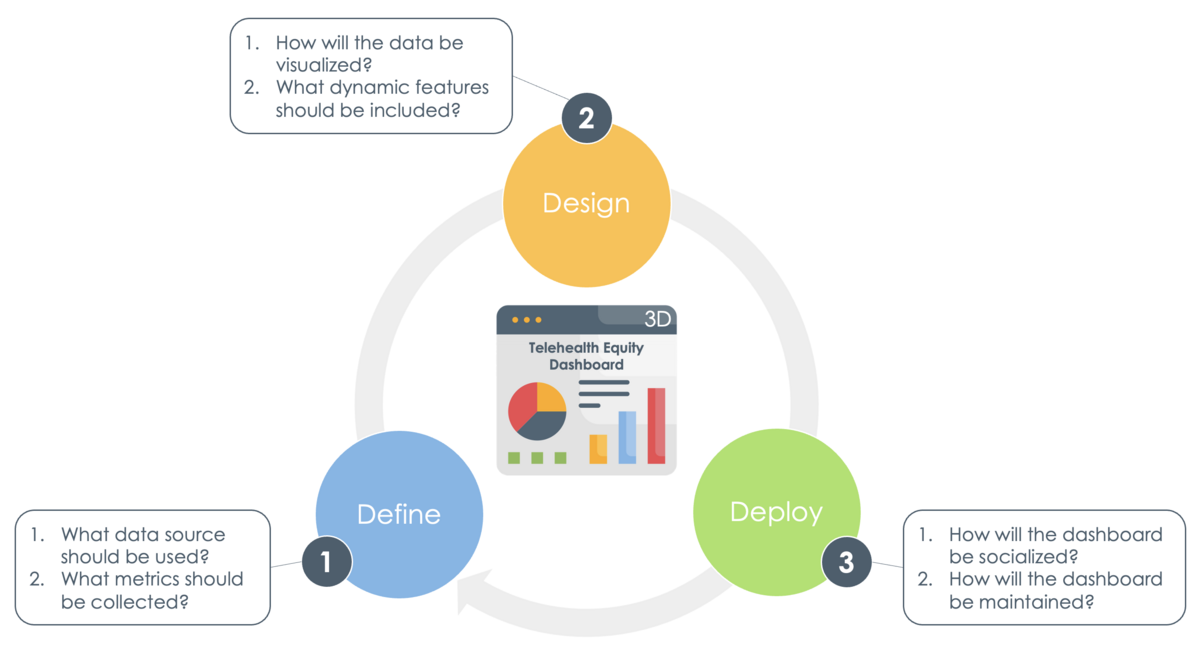
Telehealth equity dashboard framework.

**Figure 2:**
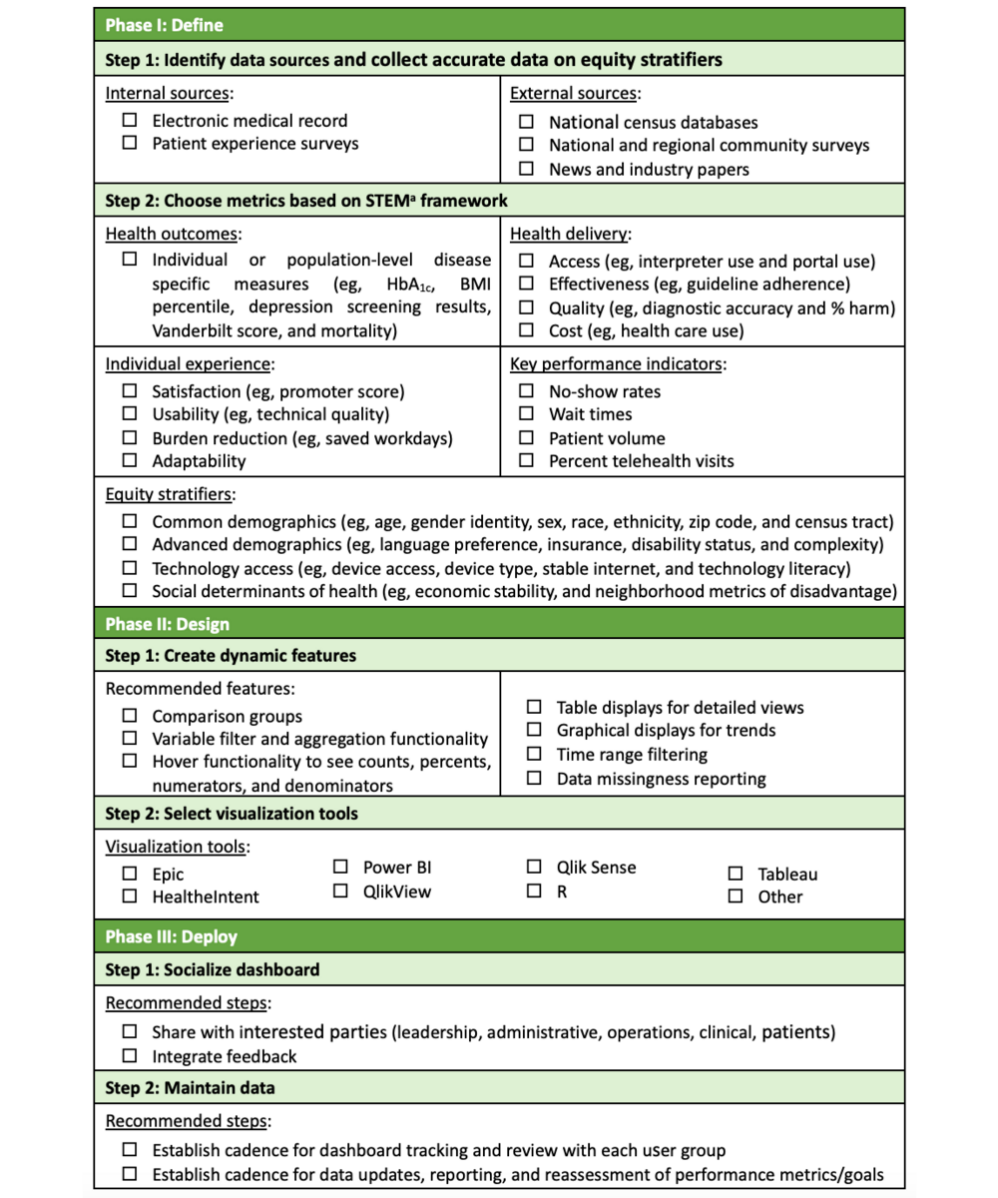
Telehealth Equity Dashboard Checklist (aSTEM: SPROUT Telehealth Evaluation and Measurement).

To standardize such dashboards, the Supporting Pediatric Research on Outcomes and Utilization of Telehealth (SPROUT)–CTSA Network formed the Telehealth Equity Workgroup. Evidence on best practices for the collection and use of equity-related data continues to evolve. Based on the review of the existing literature and the operational experience of clinicians, informaticists, and researchers in this workgroup, we aim to describe a strategic framework for adult- and pediatrics-serving health systems to execute telehealth equity dashboards through 3 phases: define, design, and deploy (Figure 1). In addition, we offer a checklist for framework navigation (Figure 2) to motivate more critical monitoring and evaluation of health systems’ current telehealth practices and ultimately identify service delivery gaps.

## Engaging Interested Parties

Before beginning to create a telehealth equity dashboard, health systems must identify all interested parties to balance diverse perspectives and priorities. This should include all potential dashboard users such as clinical staff, investigators, and administrators as well as dashboard experts and patient advocates. Early engagement facilitates institutional buy-in to both the development and use of a dashboard. In addition, as there is notable variation in data privacy regulations based on patient age, type of medical problem, local health system policy, and federal laws, early involvement of senior leadership can help ensure dashboards are implemented appropriately. Once identified, interested parties must be continuously engaged throughout all phases of the framework process to ensure these dashboards are developed with the intended users in mind.

## Phase 1: Define

First, health systems should consider what data sources to leverage. Data source mapping is one useful technique to identify usable sources for dashboard development. This inventory process involves cataloging all available sources and describing potentially relevant data to allow teams to consider the feasibility, reliability, and quality of these sources [9].

Poor data quality can have negative downstream impacts, as inaccurate or incomplete data can mask disparities [10]. First, patient and caregiver demographics can often be conflated in pediatric and elderly care settings. In addition, previous research found that non-White patients were less likely to have the correct race in their health records and were often mislabeled as White, skewing disparities [11].

Several strategies can mitigate the limitations of missing or inaccurate data [12]. Imputation or Bayesian modeling techniques can help bolster existing data by addressing missingness with inferred values. For example, imputing race and ethnicity identified greater disparities in the COVID-19 pandemic compared with only excluding missing data [13]. Health systems can also enhance existing data by linking their databases to external sources to conduct area-based monitoring [14]. To illustrate, health systems could integrate regional-level population data from national datasets (eg, the National Survey of Children’s Health or the American Community Survey for United States health systems) with internal patient data by census tract. Inequities can then be tracked between geographic regions to further support patients from medically underserved areas.

Unfortunately, these methods fail to address the root of data inaccuracy. Improvement of data collection processes is the best long-term solution. Staff training, patient education, and options for self-reporting outside of clinical encounters are the key to improved collection [10]. Greater transparency regarding the purpose of data collection and improved framing of questions to reduce discomfort in sharing sensitive data could also increase self-reporting [11].

Once data sources are established, health systems can select metrics from the domains of the SPROUT Telehealth Evaluation and Measurement Framework [8], including health outcomes (ie, disease-specific measures), health delivery (ie, quality and cost), individual experience (ie, patient experience data), and key performance indicators (ie, implementation measures), as well as equity stratifiers (ie, environmental and patient attributes). In addition, defining each metric’s performance target is critical. Targets can be based on peer organizations’ performance, past institutional achievements, national-, state-, or county-wide standards, and public policy goals.

## Phase 2: Design

Next, health systems should carefully consider the design of their dashboards, as literature demonstrates how data aggregation and visualization influence the ability to detect disparities. Common broad racial or ethnic categories such as Black or Hispanic obscure within-group differences that can have significant clinical implications [15]. For example, when Asian is grouped with Native Hawaiian and Other Pacific Islanders, such aggregated statistics conceal meaningful differences between subpopulations [16]. Thus, it is important to present data as disaggregated by equity stratifiers as possible, acknowledging that some level of aggregation is necessary given data quality limitations. A recent proposal for revised federal government standards for race or ethnicity classification may guide new best practices [17].

We recommend, at a minimum, comparing data from medically underserved populations tailored to each health system with an aggregated “catch-all” category. Health systems may consider including a reference, which is often the total population, or the group with the largest population, the most favorable health outcomes, or the greatest socioeconomic advantage [18]. However, there are risks of identifying a “reference” group. Selecting White, for example, as the “reference” population may inherently imply “nonreference” populations require assimilation or acculturation or are generally “abnormal.”

In addition, designing dashboards with filter functionality across multiple metrics can provide more robust analytics and displays. Irrespective of the population that a health system serves, intersectionality, or the connection between personal identities, is another key attribute to dashboard design, allowing for a more in-depth look at identified disparities. Race as a stratifier on its own could be a proxy for other variables underlying why these disparities exist. However, through filter functionality, users might consider assessing telehealth equity across races with another key attribute such as social determinants of health or internet access [18].

Designers should follow best practices for data visualization [19], including maximizing data-ink ratios and selecting the appropriate software for desired displays. Commercial visualization tools can be found in Figure 2. When choosing visualizations, it is essential to consider ease of interpretation and potential risks of misrepresentation. Tables explicitly lay out comprehensive information but can be difficult to digest. Interpretation can be supported through bolding or color-coding. Graphs can simplify data presentation and draw attention to specific insights, but this simplicity can be misleading [18]. It is essential to include missing data percentages to illustrate uncertainty and incorporate features to understand the context of the data for accurate interpretation. For instance, when interpreting a narrowed disparity, the availability of hover functionality to display numerators, denominators, and count breakdowns for each data point can help users understand the source of this change. In addition to reporting current statistics, the ability to view metrics over time permits the detection of trends and postintervention changes in disparities, which is an essential dashboard function.

Once a preliminary design has been determined, teams can develop a draft dashboard. From this point forward, design and development should proceed concurrently. The draft dashboard should undergo pretesting with sample end users, which can subsequently inform alterations to the design. Keep in mind, multiple designs are likely needed to accommodate different audiences, from frontline staff implementing care and monitoring day-to-day activity to administrators interested in quarterly or annual trends.

## Phase 3: Deploy

Finally, intentional deployment of a telehealth equity dashboard is critical to increase use, inform and monitor operational and clinical interventions, preserve institutional buy-in, and create a data-driven culture to improve health equity.

Socialization, the process of organizations adjusting to, learning about, and buying into a new initiative, is a key aspect of successful dashboard deployment. Socializing with leadership and clinical providers allows teams to create relationships for long-term reporting and inspires clinicians to use the dashboard in day-to-day operations. Normalizing the use of equity dashboards at all levels can stimulate maintained awareness and action to improve telehealth equity hence laying the foundation for a culture of accountability and quality data collection to address disparities in telehealth and beyond.

In this phase, it is also essential to identify a cadence of dashboard review and updates, given the likely differing preferences among users. For example, leadership may expect a quarterly update on high-level telehealth equity experience, while interpreter services may desire monthly check-ins to monitor progress on their practice changes. Socialization with regular review allows for opportunities for feedback, which studies have shown improve data quality [20]. By recognizing the appropriate set of interested parties, health systems can continue to enhance their dashboards with the right feedback from a broader and inclusive user group.

Once the dashboard has been deployed, data can be used and updated to advocate for new programs or workflows supporting medically underserved populations. The implementation of a dashboard is an ongoing, iterative process through each phase. For example, the telehealth equity dashboard may highlight a disparity that motivates the creation of a new intervention. The implementation of a new intervention may then require new metrics to be added to the existing dashboard or identify other ways to track performance. The dashboard development team may thus return to phase 1 to re-evaluate their sources and metrics. In addition, periodic usability testing by end users can allow for the identification of these key areas of improvement for subsequent iterations. This process, akin to the plan-do-study-act cycle in improvement science, can ensure the adaptability and continual advancement of a dashboard to meet the demands of a dynamic health system [21].

## Call to Action

Dashboards offer an avenue to improve data transparency. Data sharing, especially as it relates to equity, may be limited due to lack of incentives, fear of public scrutiny, or perceived opportunity costs if data are used for research by external parties [22]. However, this creates silos between and even within health systems. Data sharing has the potential to establish shared standards and cross-institutional efforts to improve health on the population level. Therefore, as technology use in health care advances, we must pay close attention to what the data are telling us, be transparent with our progress and shortcomings, and push for change in our care models to ensure equitable quality of and access to care for all patients.

## Conclusions

The COVID-19 pandemic laid bare the implications of the digital divide on health disparities. Nevertheless, telehealth continues to serve as a potential cost-effective care model and promising access point for patients with barriers to in-person services. As such, our strategic framework for developing a telehealth equity dashboard offers a valuable means to track patterns of use and outcomes to provide the evidence needed to support continued investment in an equitable telehealth offering. Telehealth equity dashboards present a promising means to build a culture of data transparency, equity-centered implementation, and continuous improvement to narrow the digital divide and improve access to care for all patients in this expanding world of digital health care.

## Abbreviations

CTSA: Clinical and Translational Science Awards
DHEF: Digital Health Equity Framework
NCATS: National Center for Advancing Translational Sciences
NIH: National Institutes of Health
SPROUT: Supporting Pediatric Research on Outcomes and Utilization of Telehealth
STEM: SPROUT Telehealth Evaluation and Measurement
